# MFG-E8 alleviates intervertebral disc degeneration by suppressing pyroptosis and extracellular matrix degradation in nucleus pulposus cells via Nrf2/TXNIP/NLRP3 axis

**DOI:** 10.1038/s41420-022-01002-8

**Published:** 2022-04-19

**Authors:** Haiwei Ma, Chenglong Xie, Zhengtai Chen, Gaolu He, Zihan Dai, Hanchen Cai, Haojie Zhang, Hongwei Lu, Hongqiang Wu, Xinli Hu, Kailiang Zhou, Gang Zheng, Huazi Xu, Cong Xu

**Affiliations:** 1 Department of Orthopaedics, The Second Affiliated Hospital and Yuying Children’s Hospital of Wenzhou Medical University, Wenzhou, Zhejiang Province China; 2 Zhejiang Provincial Key Laboratory of Orthopaedics, Wenzhou, Zhejiang Province China; 3 School of Second Clinical Medical, Wenzhou Medical University, Wenzhou, Zhejiang Province China

**Keywords:** Cell death

## Abstract

Intervertebral disc degeneration (IVDD) is a chronic age-related degenerative disease accompanied by complex pathophysiological mechanisms. Increasing evidence indicates that NLRP3 inflammasome mediated pyroptosis of nucleus pulposus (NP) cells displays an important role in the pathological progression of IVDD. Milk fat globule-EGF factor-8 (MFG-E8) is an endogenously secreted glycoprotein with beneficial effects of anti-inflammatory, antioxidant, and modulation of NLRP3 inflammasome. However, the effect of MFG-E8 on IVDD remains unclear. In this study, our purpose is to clarify the expression changes of MFG-E8 in the IVDD process and explore the role and mechanism of MFG-E8. We found that MFG-E8’s expression was reduced in degraded nucleus pulposus tissues of humans and rats as well as hydrogen peroxide (H_2_O_2_)-treated NP cells. Exogenous supplementation of MFG-E8 could rescue H_2_O_2_-induced oxidative stress, mitochondrial dysfunction, and NLRP3 inflammasome activation and protect NP cells from pyroptosis and extracellular matrix (ECM) degradation. Mechanistically, Nrf2/TXNIP/NLRP3 axis plays a crucial role in MFG-E8-mediated suppression of the above-pathological events. In vivo, we established a rat intervertebral disc acupuncture model and found that MFG-E8 administration effectively alleviated IVDD development by imageological and histomorphological evaluation. Overall, our findings revealed the internal mechanisms underlying MFG-E8 regulation in NP cells and its intrinsic value for IVDD therapy.

## Introduction

Intervertebral disc degeneration (IVDD) is the most prevalent musculoskeletal disorder and is recognized as the primary determinant of chronic low back pain (LBP) [1]. Due to its complicated process and unclear mechanism, current clinical therapeutic strategies mainly focus on alleviating the symptoms of IVDD or LBP. Compared with the annulus fibrosus and endplate cartilage, the gelatinous nucleus pulposus (NP) tissue inside the intervertebral disc is crucial to buffering the axial stress between the spinal vertebrae. Unfortunately, due to the lack of direct blood supply, NP cells always live in a hypoxic and low-nutrient microenvironment, making them sensitive to the accumulation of inflammatory factors and reactive oxygen species (ROS) [2]. Subsequently, extracellular matrix (ECM) synthesis disorder and the hypocellularity of NP cells further aggravate the intervertebral disc’s structural destruction and biomechanical instability [3]. Inflammation, oxidative stress, mitochondrial damage, ECM degradation, cell death, etc., are all typical pathological hallmarks for IVDD initiation and development [4]. However, previous studies about NP cells have focused solely on one or two pathological events, while the elaboration on their interrelationships and interactions was insufficient.

Regarding NP cell death, except for classic apoptosis, pyroptosis, as programmed necrosis, can form holes in the plasma membrane to trigger cell rupture and mediate the activation and release of inflammatory cytokines IL-1β and IL-18, which further amplify inflammation and ECM degradation cascade [5]. In human and animal NP samples undergoing IVDD, a series of pyroptosis-occurring elements such as caspase-1, GSDMD, and inflammasomes-associated proteins were found to increase or activate, indicating that intervention of NP cell pyroptosis is highly desirable for IVDD treatment [6, 7].

As a cytosolic polymerized protein complex, the priming and activating of inflammasomes are necessary for pyroptosis initiation. Concisely, after stimulation with the cellular pathogen-associated molecular patterns (PAMPs) and damage-associated molecular patterns (DAMPs), NF-κB dimer translocate to the nucleus, leading to the up-regulation of NLRP3, pro-IL-1β, and pro-IL-18. Subsequently, the cytoplasmic sensor of the inflammasome oligomerizes and recruits ASC (apoptosis‑associated speck‑like protein containing a CARD) to form a platform to activate pro-caspase-1, resulting in the cleavage and release of GSDMD, IL-1β, and IL-18 to induce pyroptotic cell death and inflammation [8]. And inflammasomes are divided into various types according to different pattern recognition receptors (PRRs) proteins, including NLRP1, NLRP2, NLRP3, AIM2, and so on [9]. However, among them, NLRP3 inflammasome, the most widely studied multiple protein complexes, are this study’s protagonist. The overproduction of IL-1β and caspase-1 caused by dysregulated NLRP3 inflammasome is mainly involved in the occurrence and development of IVDD, which is positively correlated with the IVDD degeneration score [10]. Genetic or pharmacological elimination of NLRP3 inflammasome could effectively mitigate IVDD development [5, 11].

NLRP3 is also known for its various agonists, including specific physiological and pathogenic signals such as ion flux, ATP, ROS, mitochondrial damage, etc., and exogenous pathogens. Interestingly, events such as potassium efflux, reduced ATP, and calcium influx activate NLPR3 inflammasome and further damage mitochondria, generating a vicious feedforward cycle between mitochondrial dysfunction/ROS overproduction and NLRP3 inflammasome activation [12–14]. Mechanically, thioredoxin-interacting protein (TXNIP) is defined as a pivotal bridging molecule for ROS-inspired NLRP3 inflammasome activation. TXNIP disengages from thioredoxin (TrX, a ROS-scavenging protein) during intracellular ROS overload and then binds to NLRP3, initiating the inflammasome-pyroptosis axis [15]. TXNIP is repressed by Nrf2 and maintains a low expression level under average conditions. Thus, Nrf2/TXNIP/NLRP3 axis is a promising therapeutic target for combating pyroptosis of NP cells.

Milk fat globules-epidermal growth factor (EGF) factor 8 (MFG-E8), also term as lactadherin, is a secreted glycoprotein that is widely expressed in different tissues and organs [16–18]. Previously, the prominent role of MFG-E8 was described as coordinating the removal of dying cells by phagocytes and suppressing inflammation in several neurodegenerative disorders [16]. Until recently, the inhibitory effect of MFG-E8 on NLRP3 inflammasome-pyroptosis has been elucidated in diabetic wound and kidney injury models [19]. Moreover, during the osteoarthritis process, MFG-E8 secreted by chondrocytes inhibits synovial macrophage polarization and chondrocytic senescence through NF-κB signaling [20]. And proteomics analysis of human fetus and elderly lumbar NP tissue found that MFG-E8 is closely related to age and immunoinflammatory-related IVDD [21]. Nevertheless, up to now, the potential effect and molecular mechanism of MFG-E8’s involvement in IVDD has not been validated. Our work found that the expression of MFG-E8 was decreased in human IVDD samples, rat IVDD models, and in vitro H_2_O_2_ stimulation. In contrast, exogenous supplementation of MFG-E8 could ameliorate NP cellular pyroptosis and ECM degradation by Nrf2/TXNIP/NLRP3 axis.

## Results

### The expression of MFG-E8 decreases in the IVDD process

**Fig. 1:**
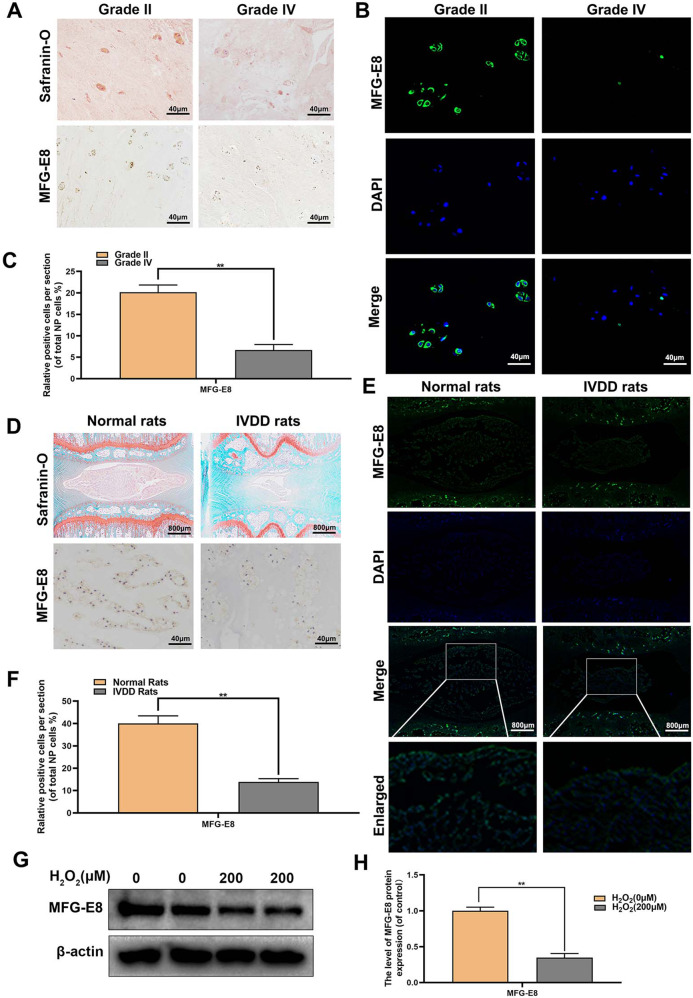
The expression level of MFG-E8 decreased in degenerated NP tissue and H_2_O_2_-treated rat NP cells. **A**, **B** Representative Safranin O staining, immunohistochemistry stain and immunofluorescence staining of MFG-E8 in human NP tissue from Pfirrmann grade II or grade IV IVDD (five sample for each group). **C** Quantitation of immunohistochemistry stain of MFG-E8 in human NP tissue (Pfirrmann grade II and grade IV). **D**, **E** Representative Safranin O staining, immunohistochemistry stain and immunofluorescence staining of MFG-E8 in NP tissue from normal and IVDD rats (*n* = 6). **F** Quantitation of immunohistochemistry stain of MFG-E8 detected in normal and IVDD NP tissue (*n* = 6). **G**, **H** The MFG-E8 protein expression was detected by Western blot in H_2_O_2_-treated NP cells (*n* = 6). All data were shown as mean ± SD. **p < 0.01.

To investigate MFG-E8’s role in IVDD development, we first evaluated the changes of MFG-E8 in human NP tissue, rat IVDD model, and oxidative-stressed rat NP cells. As shown in Fig. 1A–C, consistent with the staining results of S-O, the expression level of MFG-E8 was decreased in degenerative NP tissues (grade IV) by immunofluorescence and immunohistochemistry. Similarly, the MFG-E8’s expression level was remarkably reduced in the degenerated NP tissues of the rat IVDD model compared to the standard rat (Fig. 1D–F). The entire pathophysiological process of IVDD accompanies oxidative stress. We isolated primary rat NP cells and treated them with H_2_O_2_ (200 μM) to establish an IVDD model in vitro. Western blot analysis revealed that H_2_O_2_-induced oxidative stress reduced MFG-E8 expression in rat NP cells (Fig. 1G, H). Collectively, these results indicated that MFG-E8 expression was downregulated both locally and systemically as IVDD progresses, suggesting a potential role of MFG-E8 in IVDD pathogenesis.

### Effect of MFG-E8 on anabolism and catabolism of ECM in H_2_O_2_-treated NP cells

**Fig. 2:**
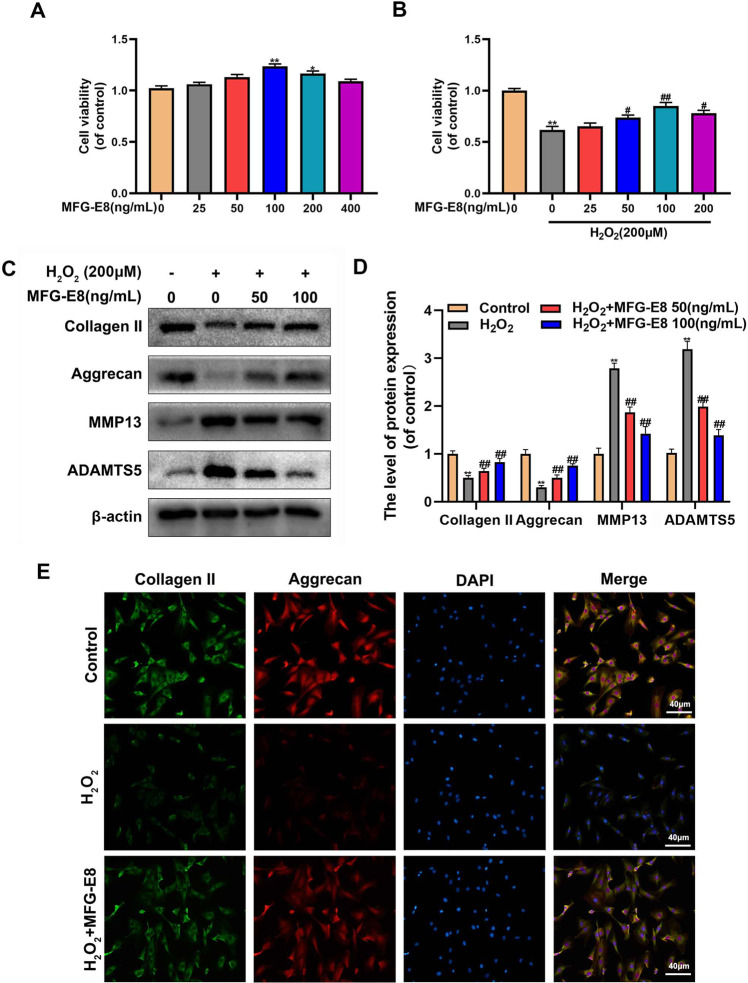
Effect of MFG-E8 in H_2_O_2_-induced ECM degeneration in rat NP cells. **A**, **B** Effects of MFG-E8 on the proliferation of NP cells with or without H_2_O_2_ exposure were measured by CCK-8 assays. **C**, **D** The protein expressions of Collagen II, Aggrecan, MMP13, and ADAMTS5 in NP cells treated as above were visualized by western blot. **E** The expression of Collagen II and Aggrecan was assessed by immunofluorescence. All data are presented as mean ± standard deviation (SD), *n* = 6; *p < 0.01 vs. untreated group, **p < 0.01 vs. untreated group, #*p* < 0.05 vs. H_2_O_2_ group, ##*p* < 0.01 vs. H_2_O_2_ group.

**Fig. 3:**
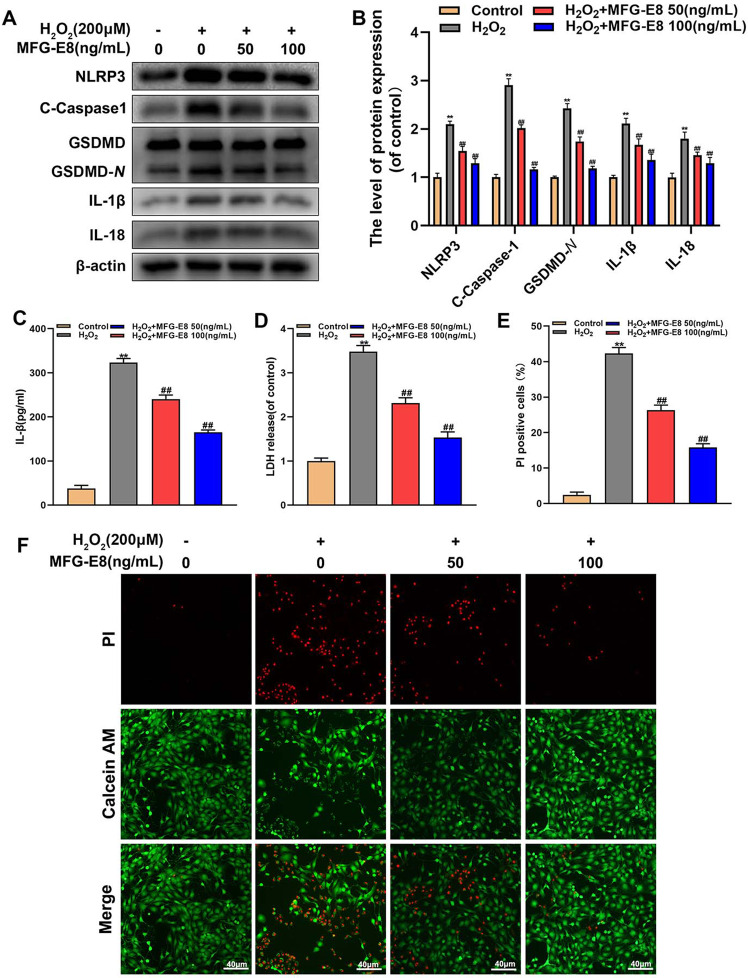
Effect of MFG-E8 in H_2_O_2_-medicated pyroptosis in rat NP cells. **A**, **B** Western blot and its quantification revealed the expression level of NLRP3, C-Caspase-1, GSDMD-*N*, IL-1β, and IL-18. **C** Effect of MFG-E8 on H_2_O_2_-exposed IL-1β production in NP cells was detected by ELISA. **D** the LDH release in NP cells. **E**, **F** Calcein-AM/PI confirmed the death level of H_2_O_2_-stimulated Rat NP cells. All data are presented as mean ± standard deviation (SD), *n* = 6; **p < 0.01 vs. untreated group, ^##^*p* < 0.01 vs. H_2_O_2_ group.

As shown in Fig. 2A, the cytotoxic effects of MFG-E8 were explored using the CCK-8 assay. The increasing concentrations of MFG-E8 treatment (0, 25, 50, 100 ng/ml) did not show any harmful impact on NP cells viability. However, there was no elevation in cellular proliferation when cultured with higher concentrations (200, 400 ng/ml) of MFG-E8 compared with the 100 ng/ml group. Additionally, we also tested the effect of different concentrations of MFG-E8 on the cellular activity of H_2_O_2_-treated NP cells. And 100 ng/ml MFG-E8 treatment showed the most protection against H_2_O_2_ exposure (Fig. 2B). NP cells degeneration is positively correlated to ECM metabolism. Herein, ECM-related proteins were investigated by western blot and immunofluorescence. As shown in Fig. 3C, D, H_2_O_2_ significantly decreased type II collagen and Aggrecan synthesis and upregulated the expression of MMP13 and ADAMTS‐5. All destructive alterations induced by H_2_O_2_ stimulation were markedly reversed by pretreatment with MFG-E8. In addition, the immunofluorescence results showed that MFG-E8 significantly attenuated the H_2_O_2_-induced degeneration of collagen II and Aggrecan. These results are strongly suggesting that exogenous MFG-E8 supplement restored the metabolic balance of the ECM in NP cells under H_2_O_2_-mediated oxidative stress.

### MFG-E8 alleviates pyroptosis in H_2_O_2_-exposed NP cells

IVDD is an aging-related degenerative disease that is potentially related to oxidative stress and NP cells pyroptosis. To make sure whether MFG-E8 alleviates H_2_O_2_-induced pyroptosis, western blot detected the levels of pyroptosis-associated proteins. Results in Fig. 3A, B showed that MFG-E8 inhibited the H_2_O_2_-caused upregulation of NLRP3, cleaved caspase-1, GSDMD-*N*, IL-1β, and IL-18 proteins. Additionally, MFG-E8 treatments reversed over-secretion of IL-1β and LDH during H_2_O_2_ exposure, based on ELISA and LDH tests (Fig. 3C, D). Furthermore, Calcein-AM/PI staining showed the MFG-E8 pretreatment reduced H_2_O_2_-mediated NP cell pyroptosis (Fig. 3E, F).

### MFG-E8 suppresses ROS production, mitochondrial dysfunction, and TXNIP-NLRP3 complex formation in H_2_O_2_-exposed NP cells

**Fig. 4:**
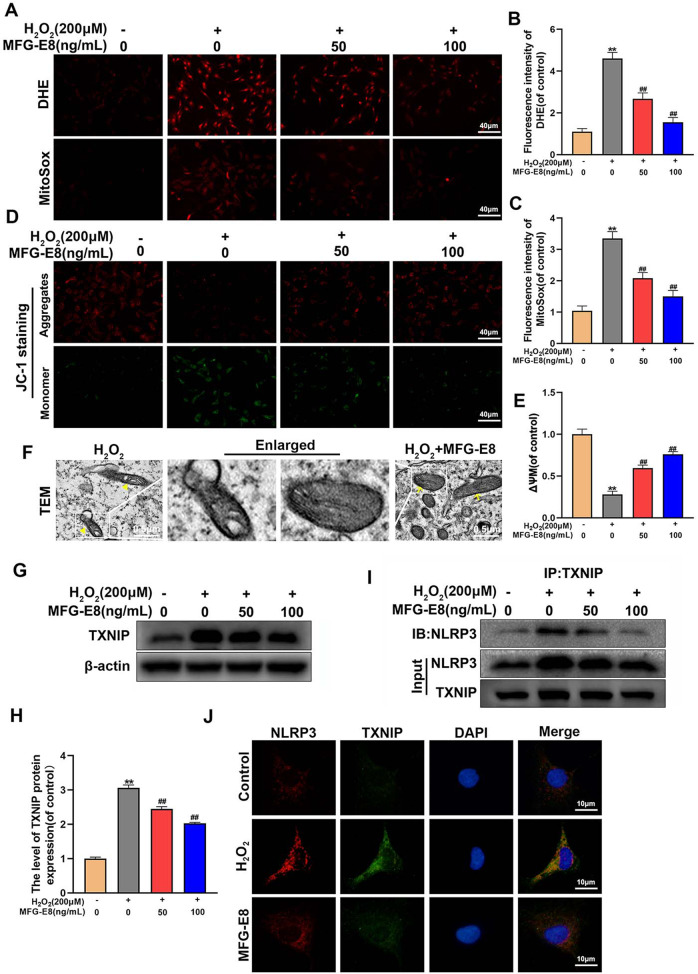
MFG-E8 suppresses ROS production, mitochondrial dysfunction, and interactions between TXNIP and NLRP3 in H_2_O_2_-treated rat NP cells. **A**–**C** Oxidative stress levels in NP cells were detected by DHE and MitoSox staining. **D**, **E** The loss of mitochondrial membrane potential in treated NP cells was measured using a JC-1 probe. **F** Observation of the mitochondrial morphology by transmission electron microscopy in NP cells (yellow triangle: damaged mitochondria; yellow arrows: healthy mitochondria). **G**, **H** Western blot and its quantification revealed the expression level of TXNIP. **I** The co-immunoprecipitation for assessing the relationship between TXNIP and NLRP3. **J** Immunofluorescence double labeled staining for colocalization of NLRP3 with TXNIP. All data are presented as mean ± standard deviation (SD), *n* = 6; **p < 0.01 vs. untreated group, ^##^*p* < 0.01 vs. H_2_O_2_ group.

Previous studies demonstrated that excessive ROS-mediated interaction of TXNIP and NLRP3 plays a crucial role in pyroptosis [5]. To confirm the protective role of MFG-E8 in ROS production, DHE and MitoSOX staining were performed in NP cells. As shown in Fig. 4A–C, intercellular and mitochondrial ROS levels were both significantly increased in NP cells exposed to H_2_O_2_, whereas MFG-E8 treatment attenuated this phenomenon. To further evaluate the mitochondrial function, the mitochondrial membrane potential was measured through the JC-1 assay, the results of which indicated that H_2_O_2_-mediated mitochondrial damage was reversed by MFG-E8 (Fig. 4C, D). And TEM results showed that the mitochondria of NP cells were damaged by H_2_O_2_ stimulation, manifested as internal vesicle formation and cristae loss. These phenomena were alleviated after MFG-E8 treatment (Fig. 4F). To investigate the effect of MFG-E8 on TXNIP/NLRP3 signaling, NP cells were treated with or without H_2_O_2_ and MFG-E8. Results showed that H_2_O_2_ significantly increases the expression of TXNIP protein, whereas this phenomenon was suppressed by MFG-E8 pretreatment (Fig. 4G, H). By CO-IP assays and immunofluorescence, we found that H_2_O_2_ stimulation enhanced the interaction between TXNIP and NLRP3, while MFG-E8 treatment reduced this effect (Fig. 4I, J). These results suggest that MFG-E8 can inhibit H_2_O_2_-mediated TXNIP-NLRP3 interaction, which is related to the regulation of mitochondrial function and subsequent ROS production.

### MFG-E8 facilitates Nrf2 signaling activation in H_2_O_2_-exposed NP cells

**Fig. 5:**
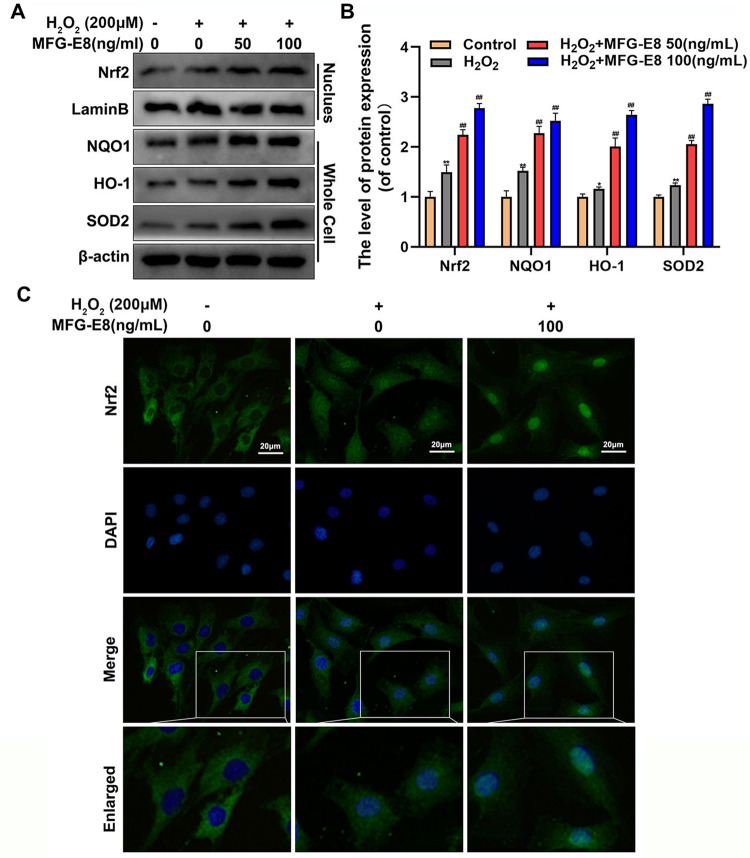
Effect of MFG-E8 on the Nrf2 pathway in H_2_O_2_-treated rat NP cells. **A**, **B** We separated the nucleus from the NP cells and evaluated the level of Nrf2 in the nucleus and the level of NQO1, HO-1, and SOD2 in the whole cells by western blot, treated with or without the administration of MFG-E8 with H_2_O_2_. **C** The Nrf2 was detected by immunofluorescence combined with DAPI staining for nuclei. All data are presented as mean ± standard deviation (SD), *n* = 6; *p < 0.01 vs. untreated group, **p < 0.01 vs. untreated group, ^##^*p* < 0.01 vs. H_2_O_2_ group.

We hypothesized that the protective effects of MFG-E8 on oxidative stress and mitochondrial dysfunction is associated with the Nrf2 pathway. Western blot results demonstrated that MFG-E8 pretreatment markedly increased the level of Nrf2 nuclear translocation compared with the H_2_O_2_ group. As expect, downstream target genes of Nrf2, including NQO1, HO-1, and SOD2, were upregulated in NP cells with MFG-E8 pretreatment (Fig. 5A, B). Nrf2 immunofluorescence staining was performed to observe the nuclear translocation of Nrf2 in NP cells vividly. As shown in Fig. 5C, the MFG-E8 group and MFG-E8 + H_2_O_2_ group possess more Nrf2 intranuclear accumulation than the control group and H_2_O_2_ group. These data suggest that MFG-E8 administration contributes to activate Nrf2 signaling.

### Nrf2 knockdown intercepts MFG-E8’s inhibition of TXNIP/NLRP3 axis in H_2_O_2_-exposed NP cells

**Fig. 6:**
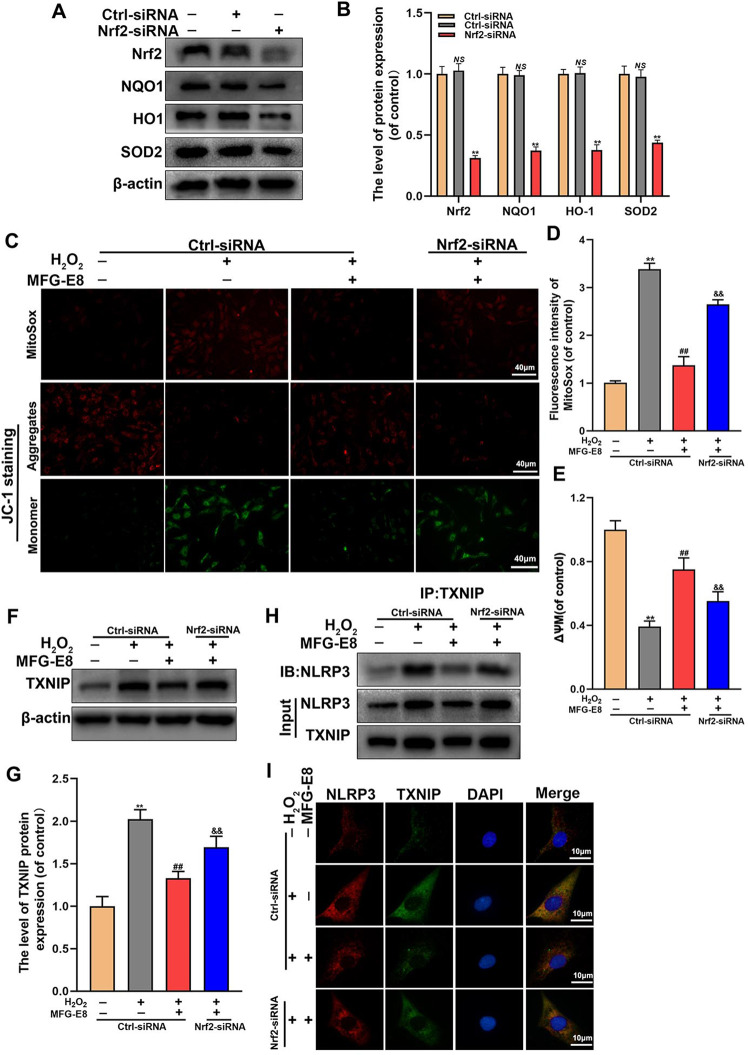
Nrf2 mediates the inhibitory effect of MFG-E8 on ROS production, mitochondrial dysfunction, and TXNIP/NLRP3 pathway in rat NP cells treated with H_2_O_2_. **A**, **B** After downregulated Nrf2 with siRNAs, the protein expression level of Nrf2, NQO1, HO-1, and SOD2 were visualized by western blot in the NP cells. **C**–**E** the ROS production and mitochondrial membrane potential loss in treated NP cells were detected by MitoSox and JC-1 probes. **F**, **G** Western blot and its quantification revealed the expression level of TXNIP. **H** The co-immunoprecipitation for assessing the relationship between TXNIP and NLRP3. **I** Immunofluorescence double labeled staining for colocalization of NLRP3 with TXNIP. All data are presented as mean ± standard deviation (SD), *n* = 6; **p < 0.01 vs. Ctrl-siRNA Group, ^##^*p* < 0.01 vs. H_2_O_2_ + Ctrl-siRNA group, ^&&^*p* < 0.01 vs. H_2_O_2_ + Ctrl-siRNA + MFG-E8 group.

It is reported that TXNIP is repressed by Nrf2 and maintains a low expression level under average conditions. However, whether MFG-E8 inhibits the TXNIP-NLRP3 pathway by regulating the Nrf2 signaling is unclear. We used siRNA to knockdown Nrf2 and the knockdown efficiency was measured by western blot. The results showed the levels of Nrf2, HO-1, NQO1, and SOD2 were markedly depressed by siRNA transfection (Fig. 6A, B). Additionally, we examined ROS production and mitochondrial function by MitoSOX and JC-1. And our results showed that Nrf2 siRNA abolished the MFG-E8-induced inhibition of ROS production and mitochondrial dysfunction under the H_2_O_2_ exposure (Fig. 6C–E). Furthermore, based on western blot, co-immunoprecipitation, and immunofluorescence results, MFG-E8-mediated inhibition of TXNIP protein expression and TXNIP-NLRP3 complex production was obstructed Nrf2 siRNA transfection under H_2_O_2_ stimulation (Fig. 6F–I). The findings reveal that Nrf2 involves the MFG-E8-induced mitochondrial protection and suppression of TXNIP/NLRP3 signaling.

### Nrf2 knockdown abolishes MFG-E8’s inhibition of pyroptosis and ECM degradation in H_2_O_2_-exposed NP cells

**Fig. 7:**
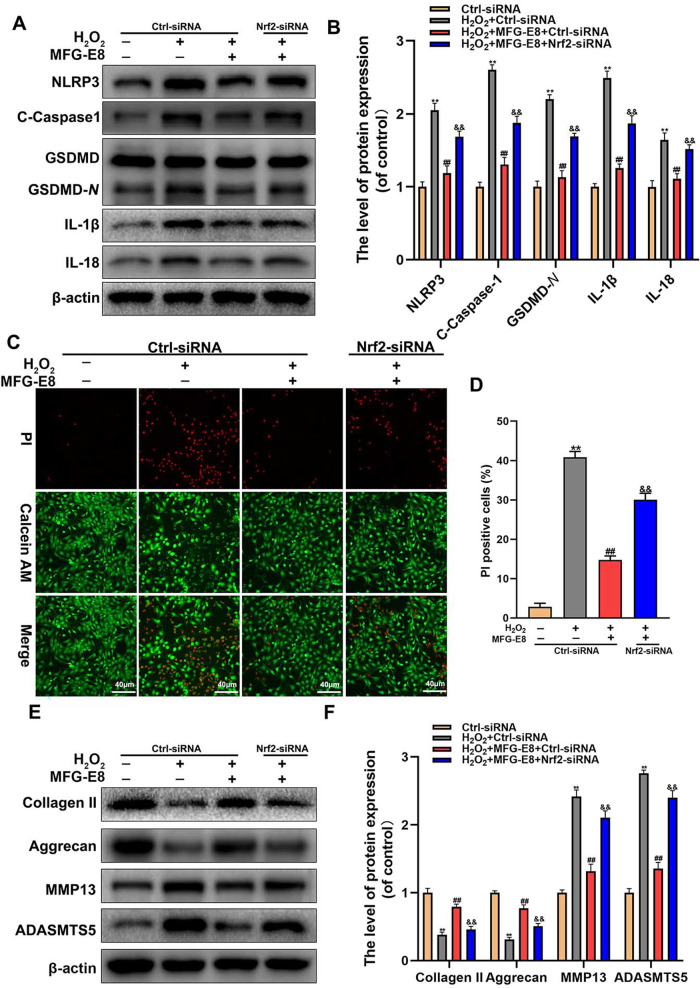
MFG-E8 ameliorates the pyroptosis and degradation of the ECM by Nrf2 activation. **A**, **B** Western blot and its quantification revealed the expression level of NLRP3, C-Caspase-1, GSDMD-*N*, IL-1β, and IL-18. **C**, **D** Calcein-AM/ PI confirmed the death level of NP cells. **E**, **F** The protein expressions of Collagen II, Aggrecan, MMP13, and ADAMTS5 in NP cells treated as above were visualized by western blot. All data are presented as mean ± standard deviation (SD), *n* = 6; **p < 0.01 vs.Ctrl-siRNA Group, ^##^*p* < 0.01 vs. H_2_O_2_ + Ctrl-siRNA group, ^&&^*p* < 0.01 vs. H_2_O_2_ + Ctrl-siRNA+ MFG-E8 group.

We further evaluated the level of pyroptosis and ECM degradation in the case of Nrf2 knockdown. Western blotting found that the pyroptosis-associated proteins were increased in NP cells after Nrf2 siRNA addition, compared to MFG-E8 and H_2_O_2_ co-treatment (Fig. 7A, B). Further, Calcein-AM/PI staining confirmed that MFG-E8 regulated H_2_O_2_-induced cell death via the Nrf2 activation (Fig. 7C, D). As for ECM degeneration, western blotting showed that the addition of Nrf2 siRNA reversed the MFG-E8-mediated upregulation of collagen II and Aggrecan and downregulation of ADAMTS5 and MMP13. In summary, the results reveal that MFG-E8 changes cell pyroptosis and maintains ECM homeostasis by Nrf2 activation.

### MFG-E8 upregulates Nrf2 activation through the PI3K/AKT signaling pathway in H_2_O_2_-exposed NP cells

**Fig. 8:**
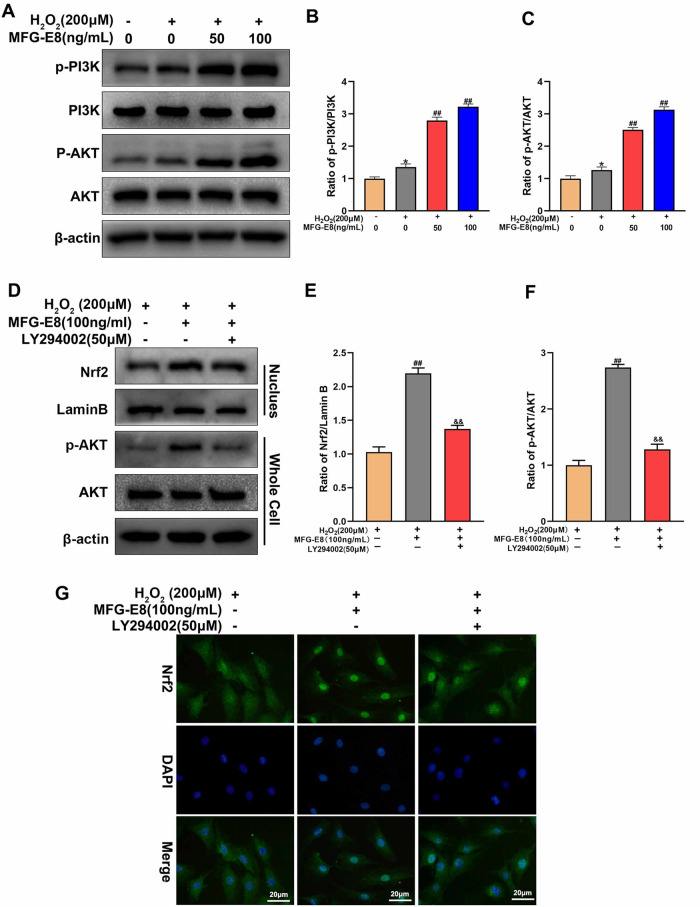
MFG-E8 upregulates Nrf2 activation through the PI3K/AKT signaling pathway in rat NP cells. **A**–**C** Western blot and its quantification revealed the protein expressions of p-PI3K, PI3K, p-Akt, and Akt in NP cells after being treated with or without the administration of MFG-E8 with H_2_O_2_. **D**–**F** We evaluated the level of Nrf2 in the nucleus and the level of p-AKT and AKT in the whole cells by western blot after in after NP cells after treatment with H_2_O_2_ or MFG-E8 or LY294002. **G** The Nrf2 was detected by immunofluorescence combined with DAPI staining for nuclei. All data are presented as mean ± standard deviation (SD) of, *n* = 6; *p < 0.05 vs. untreated group, ^##^*p* < 0.01 vs. H_2_O_2_ group, ^&&^*p* < 0.01 vs. H_2_O_2_ + MFG-E8 group.

PI3K/AKT signaling pathway, as a classic pathway in Nrf2 transcription, is also an essential pathway for MFG-E8 to play a protective role. Figure 8A–C showed that MFG-E8 pretreatment markedly increased p-PI3K and p-Akt expression. To further explore the relationship between the PI3K/ AKT pathway and Nrf2, LY294002 (50 μM, a classic PI3K/AKT pathway inhibitor) was used (Fig. 8D–F). The western blot results found that LY294002 reversed the AKT phosphorylation and nuclear expression of Nrf2 induced by MFG-E8, which was consistent with immunofluorescence staining of Nrf2 (Fig. 8G). These results suggest that MFG-E8 activates the Nrf2 pathway through the PI3K/AKT pathway.

### MFG-E8 ameliorates IVDD development in a rat puncture model

**Fig. 9:**
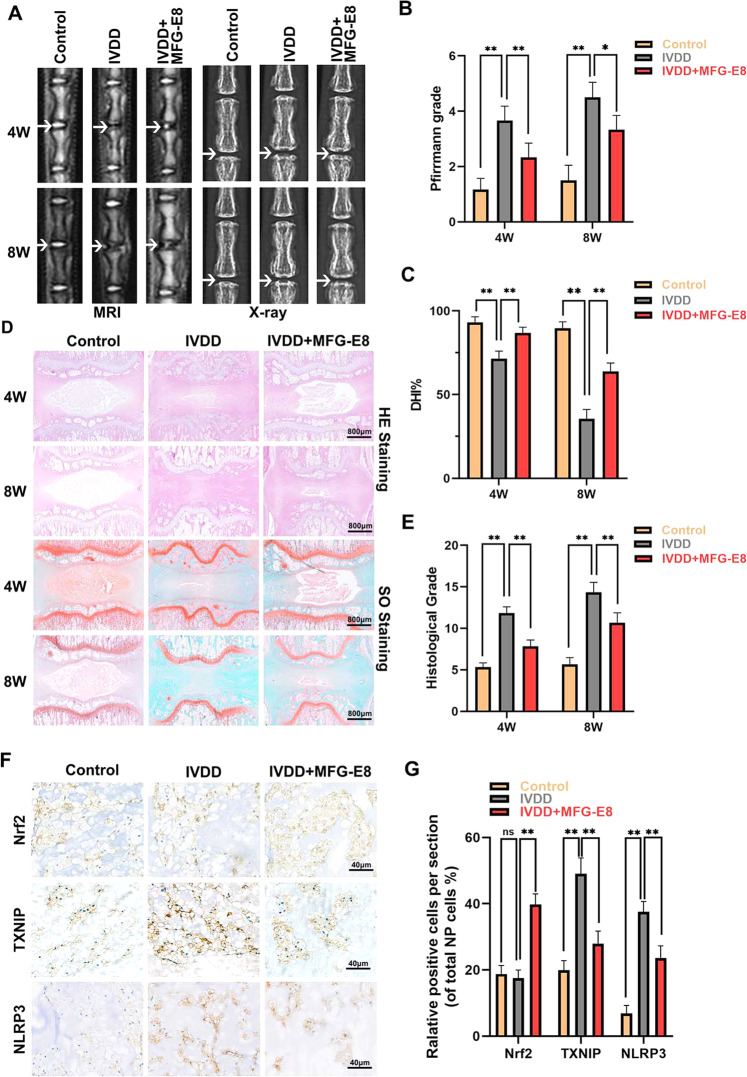
MFG-E8 ameliorates the IVDD process in the puncture-induced rat model. **A** T2-Weighted MRI and X-ray of a rat tail at 4th and 8th week after surgery (arrow: location of the needle-puncture disc). **B** The Pfirrmann MRI grade scores of the three groups at 4th and 8th week after surgery (six rats at each time point for each group). **C** The disc height index (DHI) was determined in the three groups at 4th and 8th week after surgery (six rats at each time point for each group). **D** Representative HE staining and S-O staining of punctured discs in different groups. Three sections were randomly selected for quantification, with a representative example shown. **E** The histological grades were evaluated at 4th and 8th week post-surgery in the three groups (six rats per group). **F** The expression of Nrf2 and NLRP3 was assessed by immunohistochemistry staining of intervertebral disc sections in the different groups. **G** Relative positive cells of Nrf2, TXNIP and NLRP3 were quantified by Image J. All data are presented as mean ± standard deviation (SD); *P < 0.05, **P < 0.01.

Our cellular experiments subsequently assessed whether MFG-E8 could be used for intervertebral disc degeneration therapy in vivo. Caudal discs of rats were punctured to establish the IVDD model, and X-rays and MRI were performed at 4 and 8 weeks after surgery and MFG-E8 treatment. As shown in Fig. 9A–C, the IVDD group showed a loss of disc signal on T2WI-MRI image and intervertebral height after surgery, especially in the 8th week. However, the administration of MFG-E8 partly alleviated the collapsed disc space and the loss of the MRI signal. HE and SO staining was also applied to evaluate the morphological changes of the intervertebral disc after MFG-E8 treatment. Based on histomorphology analysis, the nucleus pulposus contraction, NP cells reduction, and massive loss of proteoglycans occurred in the IVDD group while displaced by fibrochondrocyte at 4th and 8th weeks following surgery, compared with the control group. However, MFG-E8 administration alleviated these histopathological changes of NP tissues, manifested as the slight shrinking of NPs and minor loss of NP cells and proteoglycan (Fig. 9D). The effect of MFG-E8 in delaying IVDD progression was also proved by the histological score analysis from Safranin O staining (Fig. 9E). Furthermore, immunohistochemical staining of Nrf2, TXNIP and NLRP3 showed that MFG-E8 could enhance Nrf2 expression and alleviate TXNIP expression and NLRP3 inflammasome activation in the puncture-induced rat model, which was consistent with the results of our in vitro studies (Fig. 9F, G).

## Discussion

As an endogenous multifunctional glycoprotein, MFG-E8 is widely studied in neurodegeneration, tumors, wound healing and cardio-cerebrovascular disease and is even considered as a potential prognostic biomarker of vascular-aging diseases [16, 19, 22, 23]. The contribution of MFG-E8 to skeletal muscle diseases has only been reported in osteoporosis and osteoarthritis, and the mechanism mainly involves osteoclast differentiation and synovial macrophage polarization [20, 24]. Regarding the IVDD field, MFG-E8 is only mentioned in the proteomic analysis results of human NP samples and the ECM scaffolds produced by 3D culture of NP cells, whereas its role in IVDD is still unknown [21]. Our works first demonstrated that MFG-E8’s expression declined in NP cells from human IVDD samples, rat IVDD model, and in vitro oxidative stress condition, compared with the relatively normal NP cells. But interestingly, Chen et al. ‘s proteomic quantitative results showed that the level of MFG-E8 in the NP tissues of the geriatrics was 1.27 times that of the fetus [21]. These seemly contradictory results are due to differences in the selection of control samples. In this study, the NP tissue (Grade II) collected from adult patients with lumbar spine fractures as the standard group. According to the miRNA sequencing dataset GSE126677 and Professor Cai’s research, the content of miR-99 increases in synovial vesicles of patients with OA, blocking and degrading the MFG-E8 mRNA in chondrocytes, which might explain the decreased MFG-E8 in the IVDD process [20]. Additionally, in vitro cultured mouse chondrocytes and RAW cells decreased MFG-E8 synthesis and secretion after inflammatory stimulation, consistent with our H_2_O_2_-stimulated NP cells [20]. At present, acupuncture model is the most common modality for intervertebral disc degeneration models [25]. In addition to the acupuncture model, there are various models of rat intervertebral disc degeneration such as destabilization and endplate injection models [26, 27]. Compared with other models, the acupuncture model has the advantages of repeatability, simple operation and short cycle [25]. Due to the requirement for multiple intradiscal administrations, we chose the rat acupuncture model for in vivo experiments.

IVDD is a chronic age-related degenerative disease involving oxidative stress, inflammatory response, mitochondrial damage, ECM degeneration, cell dysfunction, and death. Pyroptosis is a newly discovered programmed cell death form, utterly different from apoptosis, accompanied by the amplification of inflammation cascades [28]. The core of pyroptosis initiation is the activation of pro-caspase-1, which mediates the maturation of GSDMD, IL-18, and IL-1β, and then further forms cell membrane holes and leaks inflammatory cytokines [29]. The prerequisite for cleaving caspase-1 is the recruitment and activation of the inflammasomes [8]. Although inflammasomes possess many diverse kinds, researches on IVDD mainly focused on classical NLRP3 inflammasome [5]. Our cellular experiments showed that MFG-E8 administration inhibited H_2_O_2_-induced expression of NLRP3, GSDMD-*N*, IL-18, and IL-1β and the leakage of LDH in NP cells, suggesting the pyroptosis under oxidative stress is reversed by MFG-E8. Similarly, in ATP-stimulated mouse macrophages, MFG-E8 supplementation dampened the IL-1β production and caspase-1 activity, dependent on the NLRP3 inflammasome [17]. Of course, MFG-E8 may interfere with the activation of other inflammasomes to H_2_O_2_ inhibit the pyroptosis of NP cells. Recently, Professor Yang and his colleagues found that the content of AIM2 inflammasome in human NP samples increased in an IVDD-dependent manner, which also appeared in H_2_O_2_-exposed NP cells [30]. AIM2 inflammasome were activated and damaged DNA fragments, but secretory autophagosomes also wrapped them to expel NP cells. Although there is no evidence to support that MFG-E8 could upregulate autophagy-dependent secretion, the antioxidant property of MFG-E8 have been fully elucidated in vivo and in vitro models. Hence, we speculate that AIM2 inflammasome are involved in the MFG-E8-mediated anti-pyroptosis effect for H_2_O_2_-stimulated NP cells, but further experimental verification is needed.

As the most widely studied inflammasome, NLRP3 inflammasome have multiple excitation signals during the IVDD process, such as ion flux, crystal, ATP, ROS, Propionibacterium acnes, etc [5]. In the degenerated NP tissue, the presence of hydroxyapatite crystals and calcium pyrophosphate dihydrate (CPPD) activates the lysosomal phagocytosis of NP cells, which destroys the lysosomal membrane and causes the release of cathepsin B to activate the NLRP3 inflammasome further [31, 32]. It is reported that when macrophages clear dying cells or cell debris, MFG-E8 is responsible for coordinating the fusion of phagosomes and lysosomes and acidifying the contents [17, 18]. But whether MFG-E8 also maintains the lysosomal membrane’s stability and promotes the degradation of calcium deposits is still unknown. Meanwhile, the surface receptors of the NP cell membrane convert external environmental stimuli into intracellular inflammation and necrosis signals by switching ion channels [12]. Among them, potassium or calcium flux is a standard ion change. A high concentration of ATP in the microenvironment activates P2X7 to promote potassium and ATP efflux by coordinating P2X4 and Pannexin1, causing NLRP3 inflammasome activation [14, 33]. MFG-E8, as a secreted glycoprotein, contains an EGF-like domain at the N-terminus with an RGD (Arg-Gly-Asp) module that specifically bind to integrins α_v_β_3_ and α_v_β_5_ on the cell membrane surface [16]. And Mallat et al. found that treating of macrophage by LPS upregulated α_v_β_3_ expression and the colocalization between α_v_β_3_ and P2X7 receptor, but MFG-E8 could block this interaction to counteract LPS-mediated caspase-1 activation and IL-1β release [17]. Since NP cells also express integrin protein, the above mechanism may also explain the protective effect of MFG-E8 in this study. Besides, as for the microenvironment of NP cells, acid‐sensing ion channels (ASICs) and mechanosensitive cation channels (Piezo1) regulate the intracellular calcium ion content in response to the accumulation of lactic acid and mechanical tensile stress [34, 35]. Notably, these ions current-mediated intracellular low potassium, low ATP, or high calcium could accelerate mitochondrial damage and further lead to ROS and mitochondrial DNA release, which generate a vicious feedforward cycle between mitochondrial dysfunction/ROS overproduction and NLRP3 inflammasome activation [5, 12, 13]. TXNIP is the core molecule of this positive feedback loop. When ROS is overloaded, TXNIP combines NLRP3 to recruit ASC and caspase-1 and then initiates pyroptosis [11]. Our data indicated that oxidative stress-mediated mitochondrial damage, ROS accumulation, TXNIP expression, and TXNIP-NLRP3 complex were decreased after MFG-E8 treatment. The anti-oxidative effect of MFG-E8 has been proved in LPS, or fractalkine stimulated microglia by activating the Nrf2-HO1 axis [36, 37].

Nrf2 is a crucial transcription factor for the endogenous ROS scavenging system. Under oxidative stress condition, Nrf2 transfers to the nucleus and binds with antioxidant response element (ARE) fragments on DNA to mediate the transcription of downstream genes, including HO-1, NQO1, and SOD2 [38]. TrX protein scavenges excessive ROS and locks TXNIP to form a complex to stabilize in nuclear, thereby inactivating NLRP3 inflammasome [39]. In the rat middle cerebral artery occlusion/reperfusion model, activating the Nrf2 signal reduced the cytoplasmic expression of TXNIP, resulting in a decrease in the expression and activity of NLRP3 inflammasome [40]. But these protective effects were abolished after Trx knockdown, suggesting that the negative regulation of NLRP3 inflammasome by Nrf2 is TrX-dependent [40]. In this study, we found that MFG-E8 inhibits the NLRP3 inflammasome-pyroptosis axis by activating Nrf2 nuclear displacement. Under normal circumstances, Nrf2 binds to Keap1 in the cytoplasm and is degraded by ubiquitination [41]. Furthermore, Nrf2 nuclear accumulation and transcriptional response could be carried out after phosphorylation, such as glycogen synthase kinase 3*β* (GSK 3*β*, downstream protein kinase of AKT) at the S342 and S347 site [42]. Thus, PI3K/AKT pathway inhibition promotes the degradation of Nrf2 and reduces its nuclear expression. As expect, we found that LY294002 application suppressed MFG-E8-induced AKT phosphorylation and Nrf2 activation, implying MFG-E8 regulates Nrf2 by PI3K/AKT pathway. In our vivo study, treatment with MFG-E8 enhances the expression level of Nrf2 and reduces the expression level of TXNIP and NLRP3 in the puncture-induced rat model, which was consistent with the results of our in vitro studies. Interestingly, there is no difference in the expression level of Nrf2 between control and IVDD group rats in vivo experimental. This confusion might be caused by our choice of time point at 4 weeks after surgery. At 8 weeks after surgery, the expression level of Nrf2 in the degenerate NP tissues of IVDD model rats was significantly reduced compared with control group (Fig. S1). In the early stage of rat IVDD, Nrf2 expression level may be protectively upregulated. However, its protective upregulation was not sufficient to inhibit the increase of NLRP3 and TXNIP expression level. In addition, our study found that the expression of Nrf2 was decreased along with the increase of IVDD degree, while the protein of TXNIP and NLRP3 presented a significant increase in human degenerated NP tissues (Fig. S2). These results suggested that MFG-E8 may exert its protective effects via Nrf2/TXNIP/NLRP3 axis in IVDD process.

In summary, this work first discovered the abnormal expression of MFG-E8 during the IVDD process. Exogenously supplement MFG-E8 could inhibit H_2_O_2_-induced oxidative stress, mitochondrial dysfunction, pyroptosis, and ECM degradation via Nrf2/TXNIP/NLRP3 axis. In addition, intradiscal administration of MFG-E8 delayed IVDD progression in the rat puncture-induced IVDD model. These results bring new insights into the role of MFG-E8 in NP cells and further consider its potential for IVDD therapy.

## Materials and methods

### Reagents and antibodies

Recombinant mouse MFG-E8 was obtained from R&D systems (Minneapolis, MN, USA). Hydrogen peroxide (H_2_O_2_) and LY294002 were purchased from Sigma-Aldrich (St. Louis, MO, USA). Primary antibodies against MFG-E8, Aggrecan, TXNIP, and Caspase-1 were purchased from Santa Cruz Biotechnology (Santa Cruz, CA, USA). Primary antibodies against Collagen II, MMP13, ADAMTS5, IL-1β, IL-18, NLRP3, GSDMD, PI3K, p-PI3K, AKT, p-AKT and Lamin B were purchased from Abcam (Cambridge, MA, USA). Primary antibodies against β-actin, Nrf2, SOD2, HO-1, and NQO1 were purchased from Proteintech (Rosemont, IL, USA).

### Human NP tissues collection

Human NP tissues with different degrees of IVDD were collected from patients undergoing lumbar fusion surgery owning to fracture or degenerative disc diseases. According to the MRI results combined with the Pfirrmann system to evaluate the IVDD level. Grade II disc was considered as Control group (*n* = 5, age: 45–62 years, mean = 53 years). Grade IV disc was considered IVDD group (*n* = 5, age: 50–66 years, mean = 58 years). Basic information of the patient is listed in Supplementary Table 1.

### Rat NP cells culture

Rat NP cells were isolated from young Sprague-Dawley rats (male, average weight about 100–130 g), according to the previous methods [1]. Briefly, NP tissues were collected and treated with 0.25% type II collagenase for 2 h at 37 °C. Then, the cells were cultivated for 1 week in DMEM/F12 medium containing 20% FBS also 1% penicillin at 37 °C and 5% CO_2_. Then, the culture medium was refreshed for the third time every week. The cells from the second passage were applied in the following experiment.

### Cytotoxicity assay

Cell viability of NP cells was determined using the Cell Counting Kit-8 (CCK-8; Dojindo Co.). In brief, NP cells were cultured in a 96-well plate at a concentration of 5000 per well. Then, the cells were administrated with a series of concentrations (0, 25, 50, 100, 200, 400 ng/mL) of MFG-E8 for 24 h to detect the cytotoxicity of MFG-E8. Next, the effects of MFG-E8 on H_2_O_2_-induced cell death were assessed. Following pretreatment with MFG-E8 for 1 h, NP cells were treated with 200 μM H_2_O_2_ for an additional 3 h. And the control group has only changed the medium. After treatment, CCK8 solution was added to each well and incubated for 2 h at 37 °C. The absorbance of the wells at a wavelength of 450 nm was measured by using a microplate spectrophotometer. Notably, the concentration and duration of H_2_O_2_ and MFG-E8 were based on previous studies [30, 36, 43].

### Western blot assay

Total protein was collected from cultivated NP cells or milled tissues using ice-cold RIPA lysis buffer (Beyotime) involving 1% phenylmethanesulfonyl fluoride (PMSF). The protein concentration of cell lysates was determined using a BCA analysis tool Kit (Beyotime). Next, the protein of NP cells for each sample was separated via gel electrophoresis and then transferred to polyvinylidene difluoride (PVDF) membranes (Bio-Rad, USA). After blocking using 5% nonfat milk diluted in Tris-buffered saline with 0.1% Tween 20 (TBST) for 2 h at room temperature, membranes were incubated with the primary antibodies overnight at 4 °C. Following washing three times with TBST, the membranes were incubated with respective secondary antibodies for 2 h at room temperature. Finally, the blots were detected with electrochemiluminescence reagent, and the densitometry of these blots was quantified using Image Lab V3.0 software (Bio-Rad).

### Co-immunoprecipitation (IP) assay

A commercial kit (Pierce™ Classic Magnetic Co-IP kit) was attentively used to assess the TXNIP-NLRP3 binding. According to the manufacturer’s protocols, protein lysates from NP cells were incubated with an anti-TXNIP antibody overnight at 4 °C. Then to collect immunocomplexes, protein A/G Magnetic Beads were added into the lysates, and the mixture continued rotating for another 1 h at room temperature. After washing and denaturing with immunoprecipitation buffer, the eluted proteins were immunoblotted with the anti-NLRP3 antibody through western blotting.

### Calcein-AM/Propidium Iodide (PI) staining assay

As a cell death assay, Calcein-AM/PI double staining (Beyotime) was used to quantify the number of living and dead cells. In brief, the NP cells were incubated with Calcein-AM (for living cells) and PI (for dying cells) test solution at 37 °C for 30 min in darkness. Finally, images were scanned with a fluorescence microscope (Olympus Inc), and then the percentage of dying cells to total cells was counted by Image J.

### ELISA and LDH release assay

The culture supernatants of NP cells were maintained in a freezer at −80 °C until measurement. ELISA kits detecting the level of IL-1β were obtained from R&D systems. Assays were performed according to the manufacturer’s instructions. Absorbance at 490 nm in the LDH release assays was detected on a Multiskan MK3 microplate reader (Thermo Fisher).

### Immunofluorescence

NP cells cultured on the coverslip were washed three times on PBS, followed by paraformaldehyde fixation and subsequently Triton X-100 infiltration for 15 min. Then cells were blocked with 10% goat serum albumin at room temperature for 1 h and incubated with primary antibody diluted with 10% goat serum albumin overnight in a humid room at 4 °C. The cells were washed and incubated with fluorescence conjugated second antibodies (Alexa Fluor 488 or 594) for 1 h at room temperature and stained with DAPI for 1 min. Fluorescence images of each slide were obtained under a fluorescence microscope (Olympus Inc).

Similarly, immunofluorescence staining was carried out in vivo study. After dehydrating and embedding in paraffin, the tissues were cut into 5μm sagittal sections. Following deparaffinization in xylene, sections were rehydrated with gradient ethanol for immunofluorescence. The sections were incubated in PBS containing Triton X-100 for 1 h with 10% bovine serum albumin at room temperature. Then, they were incubated overnight at 4 °C with primary antibodies in PBST. After rinses by PBS, the sections were incubated secondary antibody for 1 h at room temperature in the dark. The sections were washed three times with PBS and incubated for 10 min with DAPI before being sealed with a coverslip. Finally, images were captured with a fluorescence microscope (Olympus Inc.).

### Reactive oxygen species measurement

Intracellular ROS level was determined using Dihydroethidium (DHE) probe (Beyotime) according to the manufacturer’s protocols. After washing with PBS 3 times, NP cells were incubated in the medium with the DHE reagent at 37 °C for 30 mins in the dark. At least three images of the microscopic field were randomly obtained with a fluorescence microscope and then were measured using Image J software.

### Mitochondrial ROS detection

The level of mitochondrial ROS was measured by using Mito-SOX Red dye (Invitrogen). The NP cells were incubated with Mito-Sox Red reagent for 30 min at 37 °C, then washed three times by PBS. After being treated above, images were obtained from three randomly by a light microscope (Olympus Inc.) and then was quantified using Image J software.

### Transmission electron microscopy

NP cells were fixed in 2.5% glutaraldehyde overnight at 4 °C, post-fixed in 2% osmium tetroxide for 1 h, and then stained with 2% uranyl acetate for 1 h at room temperature. Cells were dehydrated in cold grades of ethanol and then washed three times with 100% acetone. After embedding the cells in Araldite epoxy resin, semi-thin sections were cut and stained with toluidine blue. Finally, TEM images were obtained on a Transmission Electron Microscope (Hitachi).

### Mitochondrial membrane potential (ΔѰM)

ΔѰM was assessed using the JC-1 fluorescent probe (Beyotime). Briefly, NP cells were incubated for 30 min at 37 °C with JC-1(5 μM) and then washed by PBS three times and observed by a light microscope (Olympus Inc.). For mitochondrial membrane potential (ΔѰM), the decrease ratio of red fluorescence to green fluorescence indicates a loss of ΔѰM.

### siRNA transfections

Nrf2 small-interfering RNA (siRNA) was obtained from OBiO Technology (Shanghai, China). The NP cells were seeding in Six-well plates to grow to a density of 60-70%. Following the manufacturer’s instructions, Lipofectamine 2000 siRNA transfection reagent was used with the negative control and Nrf2 siRNA. Lastly, transfection efficiency was determined using western blot.

### Animal model and treatment

A total of 36 Adult male Sprague-Dawley rats (200–250 g) were used for the in vivo experiments. The SD rats were randomly divided into three groups (including the control group, IVDD group, and MFG-E8 group). According to a published report, the rats in MFG-E8 group and IVDD group underwent a model operation [44]. The rats were anesthetized with 2% (w/v) pentobarbital (40 mg/kg) intraperitoneally. The rat tail disc (Co7/8) was located by palpation, followed by an X-ray radiograph, confirming the disc’s level on the coccygeal vertebrae. The needle (21 G) penetrates the tail skin vertically to puncture the AF with a puncture depth of 5 mm. Then, the needles were rotated 360° and then held for 1 min in the disc. After the surgery, the rats in the MFG-E8 group were intradiscal injection MFG-E8 (1 μg in 2 μL) using a 33-gauge needle, while the other group rats were administered 2 μL PBS. The volume and method of intradiscal injection were determined according to previous studies [45–47]. All injections were performed every one week until Rat sacrifice.

### X-ray and magnetic resonance imaging (MRI)

X-ray and MRI examinations were obtained at 4 and 8 weeks after surgery. After fixing the rats prone, images were acquired via the X-ray irradiation system (Kubtec, USA). The changes in disc height index (DHI) were determined using the previously described method [48]. MRI was performed to assess the signal and structural modifications in sagittal T2-weighted images (T2WI) using a 3.0 T clinical magnet (Philips Intera Achieva 3.0 MR). The MRIs were evaluated by a blinded orthopedic researcher using the Pfirrmann MRI grading system [49].

### Histopathologic analysis

After specimen collection, the specimens were decalcified, fixed in formaldehyde, dehydrated, then embedded in paraffin. Afterward, the tissues paraffin blocks were cut into 5-µm sections. Slides of each disc were stained with hematoxylin and eosin (H&E) and Safranin O fast green (S-O) staining. The cellularity and morphology of the intervertebral disc were examined by a separate group of experienced histological researchers in a blinded manner using a microscope (Olympus Inc.) [44].

### Immunohistochemistry

After deparaffinization, the sample sections were rehydrated and then blocked with 3% hydrogen peroxide for 10 min. The sections were incubated with 0.4% pepsin for 20 min for antigen retrieval and washed by PBS three times. Then, the sections were incubated with 10% goat serum albumin for 1 h at room temperature, followed by primary antibody incubation overnight at 4 °C. After being washed three times with PBS, the sections were incubated with HRP-conjugated secondary antibodies for 1 h at room temperature. Finally, at least three sections from each specimen were observed.

### Statistical analysis

All statistical analyses were performed using SPSS statistical software program 22.0 (IBM). The results were presented as mean ± standard deviation (SD) of at least five independent experiments. Data were analyzed by one-way analysis of variance (ANOVA) followed by Tukey’s test for comparison between the control and treatment groups. The Kruskal-Wallis H test analyzed Non-parametric data (Pfirrmann scores and histological grades). In case of cell experiments, *n* = xxx represents repeats. In vivo experiments, n = xxx represents the number of rat or the number of NP tissue. *P* values < 0.05 was considered statistically significant.

## Supplementary information

Supplementary Materials

## Data Availability

The data applied in support of the conclusions of this study are of access from the corresponding author upon request.
