# Supplementary material for: MFG-E8 alleviates intervertebral disc degeneration by suppressing pyroptosis and extracellular matrix degradation in nucleus pulposus cells via Nrf2/TXNIP/NLRP3 axis
Source: Cell Death Discov. 2022 Apr 19;8:209. doi: 10.1038/s41420-022-01002-8 (PMC9018842; doi:10.1038/s41420-022-01002-8)
Supplement: Supplementary file 1 — Supplementary Materials [file 41420_2022_1002_MOESM1_ESM.docx]

| Number | Gender | Age | Level | Diagnosis | Pfirrmann |
| --- | --- | --- | --- | --- | --- |
| 1 | F | 45 | L1-L2 | Lumbar fracture | II |
| 2 | M | 47 | L4-L5 | Lumbar fracture | II |
| 3 | M | 62 | L3-L4 | Lumbar fracture | II |
| 4 | F | 60 | L1-L2 | Lumbar fracture | II |
| 5 | M | 51 | L4-L5 | Lumbar fracture | II |
| 6 | M | 50 | L1-L2 | Lumbar disc herniation | IV |
| 7 | F | 54 | L3-L4 | Lumbar disc herniation | IV |
| 8 | F | 58 | L4-L5 | Lumbar disc herniation | IV |
| 9 | M | 62 | L4-L5 | Lumbar disc herniation | IV |
| 10 | F | 66 | L5-S1 | Lumbar disc herniation | IV |

**Supplementary Table 1 The basic information for each patient with different diseases**

Abbreviations: F, Female; M, Male

**Figure S1**


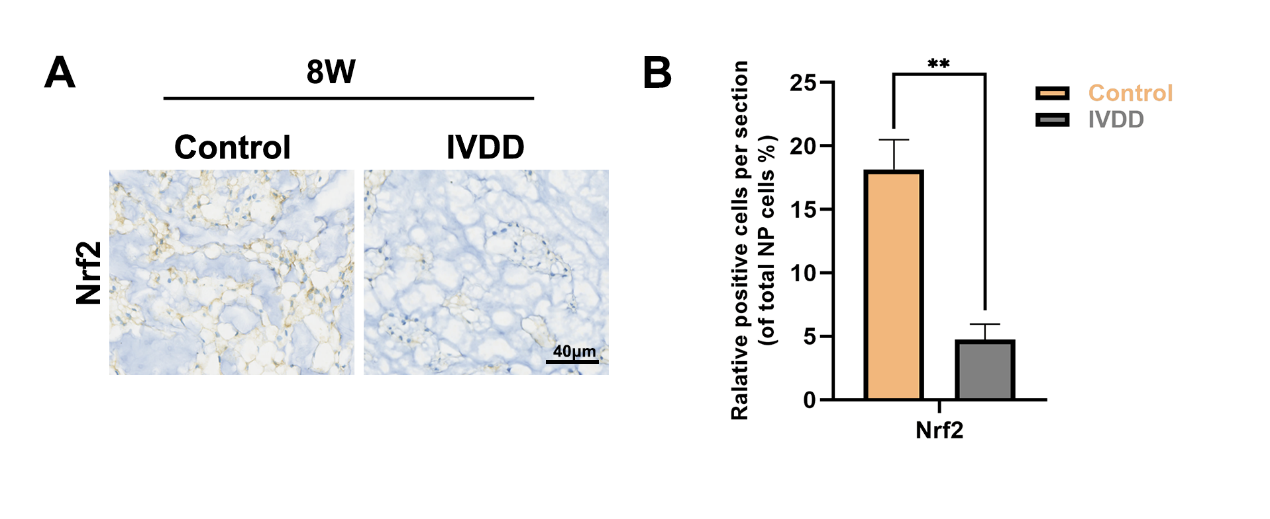


**Figure S1.** The expression of Nrf2 in rat NP tissue. (A) immunohistochemical staining of Nrf2 in rat NP tissue of control and IVDD group. (B) Quantitation of immunofluorescence staining of Nrf2. All data are presented as mean ± standard deviation (SD), n=6; **P < 0.01.

**Figure S2**


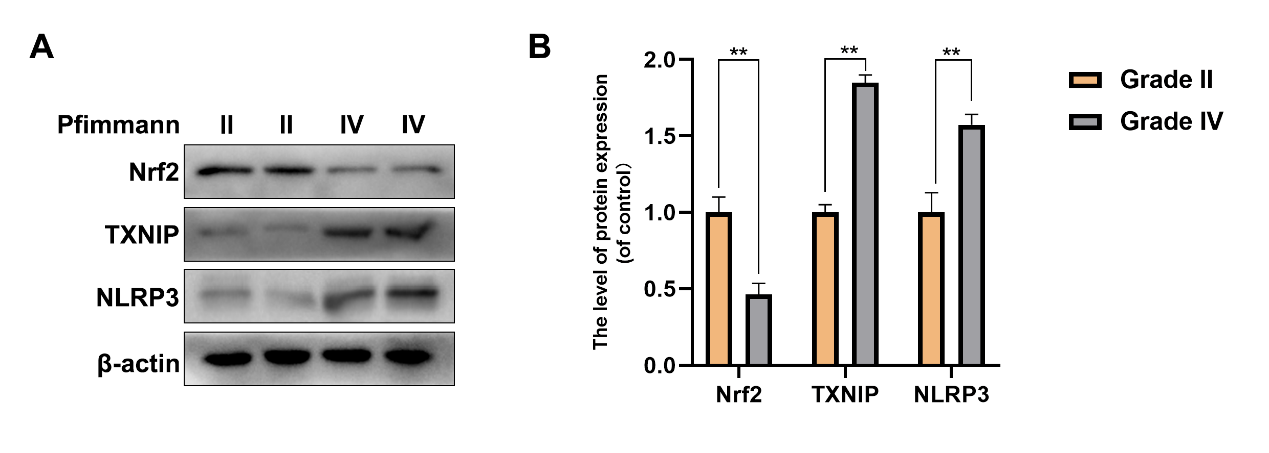


**Figure S2.** The expression level of Nrf2/TXNIP/NLRP3 axis-related protein in human NP tissue. (A-B) The protein expressions of Nrf2, TXNIP and NLRP3 in human NP tissue derived from grade II and grade IV patients. All data are presented as mean ± standard deviation (SD), n=5; **P < 0.01.
